# Perceptions on Their Own Social Participation: A Qualitative Exploration of Ethiopian Secondary Students with Visual Impairments

**DOI:** 10.3390/healthcare11040605

**Published:** 2023-02-17

**Authors:** Yisma Tsige Yeshanew, Tianxi Xu, Wei Yuan

**Affiliations:** Department of Special Education, School of Education, Central China Normal University, 152 Luoyu Ave., Wuhan 430079, China

**Keywords:** visual impairments, social participation, secondary students, disability, Ethiopia

## Abstract

Social participation is a vital part of life and has multifaceted positive outcomes on personal health and wellbeing. Social participation or the lack thereof might have more profound psychological impacts on individuals in a collectivist culture than its counterpart. The current study explored personal and environmental barriers that have hindered the effective social participation of secondary students with visual impairments. Exploration addressed various activities in and outside school settings in Ethiopia and discussed findings in relation to the prevailing cultural orientation. In-depth semi-structured interviews were conducted to gather qualitative data on barriers to social participation of 17 secondary students with visual impairments in Addis Ababa, Ethiopia. The qualitative data were analysed thematically, yielding four major themes and identifying twenty sub-themes that limited the social participation of students with visual impairments, such as personal, attitudinal, sociocultural, and practical barriers. The study showed a range of barriers that participants experienced related to social participation, the criticality of cultural orientation in providing context to understand the impacts of social participation, and the need for future research in the area.

## 1. Introduction

Social participation is key to human beings and a remedy for social isolation [1]. It encompasses an individual’s involvement in a variety of formal and informal activities [2,3] “ranging from working for organisations to inter-personal and friendship activities” [4] (p. 448). Social activities enhance quality of life, health, wellbeing, self-esteem, and social and cognitive skills [1,2,5,6,7]. The nature of the activities involved and the participant’s age determine the benefits one may obtain from social participation. For instance, while social participation helps children to develop social skills and knowledge through interactions [5], it also improves health for older adults [2,8], and significantly impacts dimensions of identity in adolescence and emerging adulthood [9].

Despite the range of advantages that social participation provides, not everyone in a society has equal access and opportunity to participate [10]. Historical accounts have shown that people with disabilities (PWDs) were generally among the socially excluded groups [11]. Some exclusionary practices included hiding, forsaking, and killing children with disabilities [11,12]. Similarly, people with visual impairments (PVIs) (including students with visual impairments (SVIs)) are commonly reported to have lower social participation and face a great risk of sociocultural exclusion from everyday life activities [13,14,15,16,17,18,19,20,21,22].

Previous studies discuss various personal and environmental factors that restrict individuals’ social participation, including functional abilities, skills, and personal interests, as well as cultural, physical, social, and institutional environments [23]. Yet, some shortcomings have also been demonstrated in the social participation literature as most research on the topic focuses on the participation of older and retired adults (e.g., [1,24,25]), or on certain activities, such as physical activities (e.g., [26,27]) in school settings (e.g., [28,29]), and in formal services. However, little attention has been paid to individuals’ participation in the community (e.g., [30]). Notably, most empirical evidence mainly concerns the conditions of PWDs in the global north, although 80% of them live in the developing world [31], especially in Africa.

Additionally, although the impacts of participation on health, wellbeing, socio-economic status, and quality of life have been well documented [32,33,34,35,36,37], the findings’ implications have rarely been explained against cultural contexts. Considering the cultural dimension is essential, especially in disability studies [38], as society’s beliefs considerably shape norms, values, attitudes, and the reality for PWDs. Culture influences social relationships and interpretations [39] and determines perspectives on what disability is and how PWDs are treated [40]. It provides context to understand PWDs’ disabilities and participation in a given society. In Ethiopia, the belief that disability is a punishment from God for one’s parents’ sinful deeds [41] may be used to justify the exclusion of PWDs [42]. Cultural beliefs also affect the early identification of and interventions for children with disabilities [43]. The theoretical resources for the cultural model of disability espouses that “disability cannot be taken as a given but that its meanings must be understood as inherently part of culture” [38] (p. 6).

Moreover, the implications of cultural orientation as an individualist and collectivist culture in studying social participation may provide additional clues on the severity of outcomes. Individualism–collectivism is one of the dimensions that help explain cultural differences [44]. In an individualistic cultural context, individuals are deemed to stand independently and have distant social relationships. In contrast, social relationships, roles, and status are the defining elements in collectivist cultures [45,46]. Ethiopia is culturally highly collectivistic, involving group obligation and relationships to subdue individual interests [47]. Subsequently, being stigmatised in a collectivistic cultural context usually has adverse relational impacts because, in this cultural reality, norms determine relationships and are enforced without explicit communication. Ethiopia, a country with a high-context (collectivist) culture, tends to have more implicit and vaguer verbal and non-verbal communications, powerfully controlling individual society members in many subtle ways [47] and impacting social interactions and participation. For those with disabilities, this situational circumstance often presents additional difficulties in hindering their meaningful communication and social engagement.

Because social life and relationships are more valued in a collectivist cultural context [46,48,49,50], the feelings social deprivation creates would ostensibly be more intense. Beyond the direct disadvantages of inadequate social resources, the meaning and value attached to social worth (in the context of collectivistic culture) can cause psychological trouble. To this effect, empirical evidence shows that culture impacts individuals’ psychological processes [51], identity, and personality [52]. This might imply that (a) cultural context highly influences the participation of exceptional groups in a society; and (b) there is bitterness of social exclusion for individuals in a collectivist culture, in which social relations, support, and interdependence are essential.

Given the critical impact of culture on the health and wellbeing of participants, the present study aimed to explore the barriers to social participation for secondary SVIs in Ethiopia. A secondary school setting encompasses more social interactions [53] and (even in the best of circumstances) is one of the most difficult settings involving intense biological and social pressure on students [54]. Given these perspectives, this study focuses on the experiences and perceptions of secondary SVIs regarding their participation in structured and unstructured social activities in and out of school settings. The findings are discussed in relation to the collectivist cultural context in which the participants are acculturated.

## 2. Materials and Methods

### 2.1. Design

This study used an exploratory multi-case qualitative design to understand complex situations contextually [55,56]. Using the qualitative method, individual semi-structured interviews were conducted with secondary SVIs. The design enabled free exploration of the complexities of the social participation phenomenon and provided insight into how SVIs experienced and perceived the phenomenon in their school and community settings while participating in formal and informal activities.

### 2.2. Participants and Settings

With the help of school officers, seventeen participants who had only vision impairments and were willing to share their experiences were selected from three inclusive secondary schools in Addis Ababa. To be able to see the impacts of vision impairment and other personal and environmental contextual factors on social participation, SVIs with additional disabilities were not included. Variations in the number of SVIs in a school setting may cause changes in the pattern of social interactions and participation. Three schools (with relatively higher and lower numbers of SVIs) were selected to gain insight into the impacts of this variation. While Schools A and B had higher numbers of SVIs (*n* = 22 and *n* = 14, respectively), School C had only two. Of the 17 participating SVIs, 5 were male and 12 were female; 6 were students with blindness, while 11 had low vision; none had any additional impairment. The students’ ages ranged from 17 to 22, with the average age being 19.88. Perhaps due to their impairments, the participants were older than typical students, on average. Additionally, while three students were impaired from birth, the remaining 14 acquired their impairment when they were between one and seven years old.

### 2.3. Procedure

This study was approved by the Human Research Ethics Committee of the authors’ university. The Addis Ababa City Administration Bureau of Education granted a letter of support to the schools. Consent letters, including confidentiality clauses and participants’ rights, were then prepared. As the participants could not read and few were under 18, proxy consents were obtained from their parents or caregivers to participate in the study and publish this paper. Interviews were conducted in the participants’ schools. Field notes were taken, and each interview was recorded, except for four students unwilling to be audio-recorded.

Focus group discussion was conducted as a pilot study to help reframe the research design, refine the research questions, and clarify the concepts and languages used in the interview protocol. Some changes included selecting the most appropriate term for vision impairment (*ayne-siwur* instead of *mayet yetesanew*) in the Amharic language and deciding to do semi-structured interviews instead of focus group interviews to avoid participants’ discomfort about disclosing personal and family-related issues observed during group discussion. The interview questions, which included participants’ demographic information and their families’ socio-economic backgrounds, were designed to explore diverse personal and environmental barriers and understand how they denied SVIs access and participation opportunities in and outside school settings. Each interview took an average of 45 to 60 min. Respondents also confirmed that their responses had been transcribed correctly and that the findings and conclusions were reasonably drawn.

### 2.4. Data Analysis

In this study, data were analysed thematically. The thematic analysis involves searching for common patterns of meaning to emerge as significant themes for describing the phenomenon [57]. Braun and Clarke’s Stages of Thematic Analysis [58] were applied in this study. The collected data were transcribed and organised in a readable format with which we became familiarised. As part of the analysis, familiarity took a long time, until the researchers better understood what was raised by whom and so on. This further involved a conscious examination of the data in terms of their relevance to the research questions. This step of data examination was followed by coding, another important data analysis stage. Coding involves identifying relevant and related aspects of the research questions [58]. Accordingly, concepts and ideas crucial to the basic questions of the research were identified from the data, categorised, and formed themes (see Appendix A, Table A1). The authors re-examined the themes, sub-themes, and codes before achieving consensus. Participants’ responses were tabulated to see the distribution of themes and facilitate using measuring words (such as a majority of, a few of … participants) to indicate the number of respondents sharing similar opinions while presenting results (see Appendix B, Table A2).

## 3. Results

**Figure 1:**
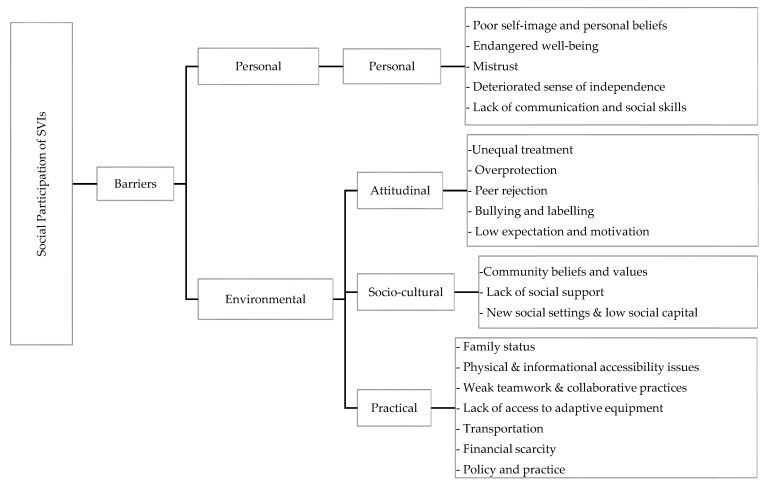
Themes and sub-themes which emerged from interviews with SVIs.

The study participants reported many similar and unique social participation experiences across settings. The prevailing cultural beliefs toward PWDs and the similarity of personal and environmental conditions may explain the resemblance. The analysis gave rise to four major themes and twenty sub-themes (see Figure 1 below). Each theme was based on significant barriers to social participation in formal (extra-curricular, community-based platforms, religious services, and events) and informal (family-based social events, community-based sport and artistic events, sociocultural ceremonies, social relationships, and hanging out with friends) activities at home, in the community, and in school settings. The emerged themes are presented as follows in Figure 1: (I) Personal barriers; (II) Attitudinal barriers; (III) Sociocultural barriers; and (IV) Practical barriers.

### 3.1. Theme I: Personal Barriers

The first theme that SVIs experienced as personal barriers limiting social participation across settings addressed students’ personal beliefs, world views, and skills. The study found that these barriers did not exist in isolation but were subject to other environmental factors, mainly cultural values. Interaction with environmental factors and their personal conditions imprisoned SVIs in their solitary zones, as detailed below.

#### 3.1.1. Poor Self-Image and Personal Beliefs

All participants discussed their personal factors limiting participation in social activities at home, in the community, and at school. In their responses, feelings of “worthlessness” were identified in various ways as a hindrance to social participation. Some participants plainly indicated that their impairments ruined their perceptions of themselves: “*My impairment is my weakness. I feel low and undeserving and then try to isolate myself*” (S13). Most participants believed they steadily developed these negative personal beliefs due to the influence of and their unyielding interactions with the social environment. They even thought that their self-image directly replicated society’s image of them. A Grade 11 student stated his anguish, saying, “*I don’t believe people will accept me. The surrounding social environment already made me believe what they do … I see the way people see me*” (S11, from School B, Grade 11). Some participants also explained that they faced challenges whenever they tried to develop a positive self-belief and new reactions. For example, one student stated, “*Sometimes when I feel good about myself and relax, someone will appear to remind me that impairment is an enemy to being equal, independent, and other similar stuff, which always makes me feel low. I often say, ‘maybe they are right*” (S15, from School B, Grade 12). These participants highlighted that whatever caused self-doubt and poor self-image would likely also affect their confidence and social participation.

#### 3.1.2. Endangered Wellbeing

The interview discussions with the students showed that most were often stressed and unhappy about themselves due to their impairment. They revealed that their lack of satisfaction affected their social lives across different settings. A Grade 12 student concisely stated, “*I don’t feel happy due to my impairment… I don’t feel psychologically well and often prefer to stay alone as a result*” (S13, from School B, Grade 12); “*I also feel insecure and threatened when I think about the social world*” (S7). Not surprisingly, participants indicated that due to their enormous daily challenges because of their impairments, they were in constant conflict with their inner peace, further influencing their social participation patterns. The data indicated that because most regularly experienced stress and their subjective wellbeing was threatened, SVIs often lost the enthusiasm to have a more comprehensive social network and participation in and outside the school settings: “*Going out and meeting people needs positive energy and feelings. How come you think in the first place to intrude into other’s boundaries while you feel low*?” (S11, from School B, Grade 11).

#### 3.1.3. Mistrust

The interview results with SVIs showed that most were sceptical of some relationships. They reported that prior experiences of some people’s misbehaviours affected their trust and social participation. One participant concisely said, “*Some people take advantage of our vision loss in the name of support. I’ve been deceived a few times*” (S5, School A, Grade 11); “*I experienced theft and harassment on different occasions. Going out always has consequences*” (S8). In addition to material deceptions and other physical crimes, two female participants reported experiencing sexual abuse, which ruined their social trust: “*A few weeks after I came here [to the capital], someone whom I haven’t recognised yet tried to rape me, pretending that he would help*” (S9, School A, Grade 12). This and other abuses and misconduct altered the trust SVIs had, especially in new people, impacting their social participation and independent movement: “*Could you imagine someone who has sight will steal your white cane? … But, this happened to me. That sort of experience spoils my social life in general*” (S4). Most participants highlighted the risk of finding themselves in strange places with strange people due to their fear of multiple types of crimes, directly impacting their mobility and participation.

#### 3.1.4. Deteriorated Sense of Independence

Interesting views were reflected regarding the need for independence in the existing social context of Ethiopia. While some acknowledged the risk of being independent, others expressed concern that their lack of autonomy hindered social participation. Participants who doubted the importance of independence for participation mentioned the risks (raised above) of being independent and the nature of social life in Ethiopia: “*We are culturally interdependent. Deviating from the group and standing alone would give a hard time instead*” (S10). However, these participants did not deny the essentiality of independent life skills, such as independently participating in self-caring. Participating students who highly favoured independence as social enablers explained that their lack of skills to manage their everyday life independently affects their social participation: “*There are times you need to do things on your own. Seeking out others’ support will restrict your reach*” (S8, School A, Grade 12). This same student noted that failure to do things independently inevitably causes feelings of shame which impacts the enthusiasm to participate in a social gathering. Additionally, difficulties in identifying places, travelling alone, and making independent decisions were reported to hinder participation. One student said, “*The need for independence skills begins when you wake up and never ends even after sleeping. You may learn some of these skills, but others will still affect your daily activities, including social participation*” (S5, School A, Grade 11).

#### 3.1.5. Lack of Communication and Social Skills

Most participating SVIs revealed a lack of interpersonal and social skills, which rendered them socially isolated. For these participants, such skills are compulsory for social engagement. They categorised their personalities as ‘shy’ and ‘introverted’: “*I don’t have the know-how or experience to introduce myself and initiate a discussion with someone who is not intimate*” (S3, from School A, Grade 9). Some participants did not mention that such difficulties might have their roots in the past. In the interviews, most participants stated that many social problems accompanied their childhood. The data revealed that almost all participants experienced loneliness during childhood as their parents hid them in fear of social pressures: “*I rarely met and played with peers in my childhood, which may have deprived me of learning some skills*” (S8). Similarly, all participants who experienced vision impairments from birth (S3, S9, and S13) and ten other participants who acquired the impairments at age three or younger indicated they had few chances to play with peers during childhood. According to these participants, this experience resulted in, among other things, poor communication and social skills—which were reported as crucial skills for social participation. Participants believed that the sociocultural climate did not provide fertile ground to ensure personal growth and considered it the reason they were denied opportunities to learn basic skills during their formative age. The lost opportunities are now underlined as they are crucial social roles for a young adult.

### 3.2. Theme II: Attitudinal Barriers

Unfavourable attitudes across settings caused massive damage to SVIs. Participants unanimously reported that attitudinal barriers considerably limit their social participation. They noted that barriers pertaining to attitudes toward them vary in their nature.

#### 3.2.1. Unequal Treatment

Most students in this study reported unequal treatment and privilege across settings compared to their siblings and sighted peers at home and beyond. One student commented, “*Even my parents favour my siblings both in material and non-material aspects. Once I was told that I only needed a few things as I had little mobility compared to my two brothers*” (S1, School A, Grade 9). Most participants also reported having fewer opportunities to participate in family social networks: “*Physical presence doesn’t guarantee equal treatment, which is worse than isolation*” (S14). Most participants disclosed that parents, neighbours, and teachers vehemently favoured sighted children and seemed to agree on the primary cause of such maltreatment: “*People do not behave the same way if this is not where we’re culturally positioned*” (S6).

#### 3.2.2. Overprotection

Overprotection was reported and various other relevant factors impacted their social participation, including independence, skill learning, and relationship formation. Participants also mentioned that overprotection by family and caregivers, especially during their younger years, was a barrier to learning social skills and establishing social networks: “*I’ve rarely been left alone whenever I’m out of my home*” (S17). Most participants agreed that overprotection hampered their social participation and facilitated social exclusion. Some SVIs, such as S5 and S6 (S5, from School A, Grade 11; S6, from School A, Grade 11), felt suffocated and manipulated when they were overly protected. According to them, parents tried to control every move they made for diverse reasons, including fear of accidents and stigma. One student said, “*Even if I’ve changed, I’ve still been protected [by my parents] like I used to be a decade ago. I can’t hang out with friends*” (S6, School A, Grade 11). Many students still living with their families said that overprotection was an attitudinal enemy to social participation.

#### 3.2.3. Peer Rejection

In school, SVIs reported having distant relationships with peers, teachers and the school community, often experiencing rejection by their peers: “*Rejection can be common to everyone, but the most common experience to us, everywhere by everybody*” (S7). Most students expressed dissatisfaction with their relationships with the school community, particularly with peers. According to one Grade 11 student in School B, “*Peer rejection is common. Sighted peers perceive us as dependent and leave us alone*” (S12, from School B, Grade 11). According to these participants, a lack of interest on the other end of the relationship substantially impacted their social relationships and participation. Some participants experienced sharp pain due to peer rejection: “*Rejection is denying a relationship and social space, an insult that disgraces the rejected*” (S14). Many of these participants were excommunicated and experienced rejection at least once in their life.

#### 3.2.4. Bullying and Labelling

Bullying and labelling were commonly reported barriers to SVIs’ social participation in schools. Most participating students encountered these challenges repeatedly; “*For me, bullying alone can lead to self-isolation; it’s painful*” (S17). Data showed that these problems increased in secondary school. Participants revealed that some sighted students worked hard to make fun of them, which is detrimental to their social participation in school. Regarding these school phenomena, a student said the following:

*“I finally discovered that I had a nickname used by sighted students. I tried to reach the name giver but couldn’t. I reported to the school, but in vain. I have no option other than learning with students who don’t call you by your name”*

(S9, from School A, Grade 12).

These and other experiences of bullying and labelling were mentioned in association with ostracising environmental factors. Keeping oneself away from participation was seen as a solution.

#### 3.2.5. Low Expectations and Motivation

Most participating students revealed that low expectations and motivation significantly limited their participation. This challenge, according to participants, has its root at home and elsewhere: “*Some people put you down even in tasks or activities that do not necessarily require visual ability. They will jump over you and invite others instead. Teachers, peers, parents, siblings, neighbours all do this thing*” (S12, from School B, Grade 11). Those students who mentioned low motivation and expectation as social environments disabling participation made it clear that spending time with people who viewed one as a quiet achiever was painful: “*Some people will tell you that this is not for you... It’s an insult that you are incapable… Such tendency loosens social networks*” (S17, from School C, Grade 10). Participants showed that this circumstance eroded their interest in companionship and weakened participation.

### 3.3. Theme III: Sociocultural Barriers

#### 3.3.1. Community’s Beliefs and Cultural Values

One of the most problematic areas for SVIs’ social participation rests on the social value system. Participating SVIs unanimously agreed that community beliefs and cultural values are among the most critical barriers to social participation and the primary source of the other challenges they encountered: “*The very reason behind our miserable childhood lies in our culture. With the culture, the misery still follows us*” (S1). Participants revealed that viewing blindness as a punishment from God was a prevailing belief and reported continuous suffering. One participant delineated, “*Some people see us as a reminder of finding a reason to praise God as a result of not being [created] blind like us*” (S8, from School A, Grade 12). This belief was referred to as a boundary that made SVIs feel alone and was a deterrence to social participation.

#### 3.3.2. Lack of Social Support

Most participants revealed that a lack of social support was a crucial barrier to widening social networks and participation. Some students reported rarely receiving psychosocial support from all but a few friends and family members. Participants agreed that non-material support does not cost schools or the community extra resources but remains scarce regardless of its benefits, further presenting challenges to social relationships: “*If there is no one to encourage and make you feel comfortable, you are already isolated*” (S2, School A, Grade 9).

#### 3.3.3. New Social Setting and Low Social Capital

Some participants raised the point that moving from one place of residence to another and changing schools practically limited social networks and caused estrangement. According to them, such a limitation hinders meaningful social participation. As the participating students’ demographic information indicated, most migrated to the capital in search of opportunities, including schooling. According to them, this circumstance prevented them from finding friends in their neighbourhoods and schools. A Grade 9 student (S3, from School A, Grade 9) said, “*One of my major problems since I came here [Addis Ababa] is to find people I know well. I’m still viewed as a stranger in the city and know limited friends*”.

### 3.4. Theme IV: Practical Barriers

#### 3.4.1. Family Status

Most participants revealed that their low family socio-economic background contributed to their reduced social participation. Most came to the capital due to poverty and a lack of opportunity in other cities. Additionally, for those who lived with their families, social participation was not an accessible business. Families were usually busy making ends meet and were less concerned with their children’s social needs. Some indicated that leisure activities cost money and time, which their families always lacked. A student added, “*My family must sweat to ensure our daily subsistence. No one cares about issues of this kind [participation]. I don’t even think that it’s important as compared to our pressing problems*” (S6, from School A, Grade 11).

#### 3.4.2. Lack of Access to Adaptive Equipment

Students revealed that essential assistive equipment—such as a tactile map, compass, flashlights, sunglasses, and a long cane—is very scarce: “*I only know a list of assistive equipment by name while some of my friends talk about them. They are far from my daily life*” (S12, School B, Grade 11). Most students clarified that their families could not provide such materials, and support from other bodies was limited. According to these participants, the absence of such equipment restricted social participation. One student noted, “*Some equipment facilitates mobility and independence. The lack of them would have an adverse impact not only on social participation but also daily lives*” (S17, School C, Grade 10).

#### 3.4.3. Physical and Informational Accessibility Issues

Nearly all participating students reported that the physical environment in classrooms, schools, and the community presented immense difficulties in mobility and participation. Most participants indicated that moving from place to place without support was challenging in classrooms, schools, and outside schools: “*It’s impossible to move and use facilities in school independently freely*” (S15, School B, Grade 12). Participants reported that uneven grounds, open ditches, wells, and roadblocks limited their mobility and social relationships. According to such SVIs, even classrooms lacked the proper arrangements of chairs and other items needed to make mobility free and without difficulty. The interview data indicated that beyond limiting SVIs’ mobility and social participation, physical barriers in and outside school caused multiple bodily injuries.

Most participants reported that access to relevant and timely information helped them remain included. Information, in this case, refers to evidence that SVIs can get from various sources in every possible way, including print, online, electronic, and word of mouth. They agreed that information is a method and resource to explore the environment. However, these students indicated they did not receive information, raising their dependency level across settings and hindering participation in some social activities. One student added, “*I missed attending some free-of-charge leisure activities due to inaccessible information both in school and the community*” (S10, School B, Grade 11). When essential information was communicated visually through reading and writing, SVIs had to seek information from their sighted peers.

#### 3.4.4. Weak Teamwork and Collaborative Practices

Findings revealed that teamwork is poorly practised in all schools. Participants described a pre-2018 collaboration practice in which every primary and secondary public school organised students into five-member groups. In addition, a student leader was often selected within each group, with the responsibility to help other members, head the discussions, and report the progress of group members. This system was said to be interrupted following the country’s political reform in 2018, as explained during the data collection.

Most SVIs highlighted the absence of alternatives or practices to encourage teamwork and collaboration. One student said, “With all its weaknesses, the former practice created a chance for me to meet peers and know each other. But there’s no such thing now to keep us networked” (S7, School A). Similarly, another student mentioned the social and academic benefits of teamwork: “Teamwork practice helped me to get support. Peers in the group tried to explain what I did not understand during classroom discussion. It was also social as we became friends in the process” (S11, School B). The results showed that the absence of such practices prohibits chances to connect with peers.

#### 3.4.5. Transportation

Transportation in the capital was reported to be very difficult for SVIs. According to participants, the transportation system deters mobility and participation. Students revealed that losing one’s personal belongings on public transport was very common in the city. Moreover, for the study participants, buying a ticket and getting on and off the city buses (the type of transportation they regularly use) were terrible experiences. Most participants agreed that such inconveniences negatively impacted SVIs’ mobility and social participation.

#### 3.4.6. Financial Scarcity

Most participants explained that financial resources were significant barriers to social participation. Some SVIs lived independently, struggling financially to make ends meet. They did different things to get money, including selling tickets to the national lottery agency (see the interview transcript of a student in Appendix A). Those SVIs were always busy with their survival and overcoming financial hardships to cover their expenses. Due to this, they found seeking additional support to meet social and special needs acceptable. They believed that accessing additional financial resources improved mobility, independence, and social participation. However, the findings showed an acute shortage of financial resources needed to strengthen SVIs’ participation and life trajectory across settings.

#### 3.4.7. Policy and Practice

Participants reported that poorly executed policies and legislation, as well as institutional malpractices, hindered SWDs’ social participation. The government’s failure to effectively execute legal and policy provisions and supervise their status was reported as a significant barrier to SVIs’ social participation.

*“You see, to say ‘the sky is the limit’ on policy documents is not a big deal. Everybody can say that. To implement them and provide limitless opportunities is the one that makes the difference.”*

(S11, Grade 11, School B).

Moreover, some participants indicated that malpractice, including physical and informational inaccessibility, lack of courtesy, differential treatment, etc., in public institutions and services, such as media, presented multiple challenges to images of and attitudes toward SVIs, further hurting their social participation.

## 4. Discussion

Ethiopia is culturally collectivist [47], and maintaining relationships is highly valued [50]. In a collectivist culture, emotions are linked with assessments of social worth and connection—not with individuals’ inner world and subjective self, as in individualist cultures [46]. Culture influences personality [50] and is crucial to shaping interactions and behaviour and informing decisions [59], which are all critical to social participation. An individual’s worldview (which is hugely culturally formed) determines preferences for participation [60]. Thus, culture provides a lens through which individuals view themselves and others, which is crucial to social engagement, especially in a collectivist cultural orientation.

Most participating SVIs developed self-doubt in many areas of their lives, affecting their social networks and participation at home, school, and community. Although negative self-perception and its behavioural impact among young people with vision impairment has research evidence [53], in a collectivist culture—where social adjustment and interdependence within groups are eminent [50]—the severe impact of social exclusion has been rarely discussed. The visually impaired participants’ assumptions about how others would perceive them and their attendant feelings of embarrassment limited their participation in sports and arts activities [60]. In a collectivist cultural orientation, emotions are emerged, embedded, and reflected as relational phenomena [46], and the kinds of relationships one has influence emotions and attendant actions. In this case, self-perception in a culturally collectivist society was affected by relational feedback, which further impacted other areas of life, including social participation. Poor self-esteem [61,62], insecurity, stress, and life dissatisfaction severely hindered social participation. Stress is negatively associated with participation in social and leisure activities [63]. SVIs’ low level of independence was reported to affect their social participation as a personal barrier. In a collectivist culture, people generally are less independent and likely to maintain the will of in-groups [49]. The fact that social interdependence is a defining element of a collectivist culture suggests that visually impaired people’s lack of independence might not only be associated with their impairment [49]. Individuals in such contexts prefer maintaining relationships to acting and accomplishing competitively [48,50].

The community’s negative attitudes toward PWDs in Ethiopia, including those with vision impairments, has earlier research evidence [64,65,66]. Multiple studies show that students with special needs experience more negative attitudes in school settings than those without impairments. Harassing behaviours and bullying of students with impairments by their non-disabled peers are among the commonly reported negative attitudes affecting social participation at school [67,68].

The influence of culture on forming attitudes, opinions, and personalities has been well documented [50,69,70], and non-disabled peers, teachers, family, and community members might learn to behave as they do toward SVIs through socialisation since childhood as normative behaviour. Notably, people with collectivist values tend to conform to the existing norm, as members are relationship-oriented and value group harmony [48,50]. Low levels of expectation and motivation in SVIs contribute to their social disengagement as attitudinal barriers. Similar findings also show the positive association between PWDs’ levels of expectation and confidence [71] and the latter’s impact on social participation [61,62].

In this study, community, societal, and cultural values remarkably restricted SVIs’ social participation. In this regard, Rao et al. [72] noted that variations in stigmatising attitudes across different cultures might be interpreted through cultural characteristics. Participants highlighted how misconceptions about disability as a punishment for sin limit their social participation. Other studies’ findings reveal that erroneous community beliefs and values are the most daunting social challenges to PWDs in Ethiopia [73,74,75]. Unlike in an individualist culture, where disability is considered a physical and individual phenomenon and a chronic illness seeking remedy, in a collectivist culture, it is viewed as a spiritual and group phenomenon that must be accepted [76]. It is reasonable to assume such perspective differences might influence other areas of life activities for PWDs, including social participation.

This study’s findings indicated that SVIs suffered from a lack of social support across settings, which hugely impacted their participation, echoing previous studies that found that poor social support is an environmental factor that adversely impacts social participation [63,77]. Support, particularly from peers, is crucial for enhancing self-esteem and social acceptance [78]. As social support and relationships are more valued in a collectivist cultural context [46,49,50], SVIs’ resultant feelings due to rejection and isolation might be severe. This suggests that more research is needed to understand what it feels like to be socially marginalised in different cultural orientations.

Family socio-economic conditions put SVIs at elevated risk of having restricted social participation. Family status is a crucial predictor of social participation in impaired youth [63,79,80]. It is significant to note that due to the financial, psychosocial, and physical burden of caring and assisting disabled family members, families often encounter multiple limitations [81], adversely impacting caretakers’ social participation.

The lack of accessible information about leisure activities and events hindered SVIs’ social participation. A previous study also indicated that inaccessible communication and information systems hindered participation opportunities [82]. This study provides evidence that physical inaccessibility and adjustment difficulties are significant practical barriers to SVIs’ social participation across settings [83].

This finding reveals that due to their design and construction, indoor and outdoor facilities—such as public institutions, transportation services, and footpaths, among others—are difficult to access for people with visual impairments in Ethiopia, negatively impacting social participation. The data also showed that transportation is a major practical barrier hampering mobility and participation in Addis Ababa. In support of this, the literature reveals that access and transportation facilities play critical roles in social participation [77].

SVIs’ lack of social and adaptation skills practically limited their social participation. Supporting this finding, another study revealed that children with vision impairments experience difficulty in forming and maintaining relationships [18,84]. Results of previous studies indicate that assuming social skills determines children’s acceptance [83], social engagement, and participation [18], and its deficits lead to exclusion from social and educational activities [85]. As cultural characteristics influence children’s social skill development [17], concerns should be taken regarding the multifaceted impact of underlying cultural values on relevant skill development and participation.

The current study reveals that poor policy implementation and institutional malpractice negatively affected SVIs’ social participation. Introducing unexecuted policies regarding PWDs alone has brought no change in Ethiopia [86]. Similarly, the findings showed that institutional malpractice in providing appropriate service for PWDs limited participants’ opportunities to participate. Prior assessments in the context of Africa revealed that cultural perspective influences perceptions of disability and shapes the kind of services delivered in Africa [40]. Hence, cultural norms affect the pattern of participation or perceptions toward PWDs and the services provided, including early identification and rehabilitation [43].

The major limitation of this study is that it is based on scarce information generated from a small sample of participants, which may raise questions about the findings’ generalisability. Consequently, future research should consider gathering additional data from more participants (SVIs and stakeholders) from school and community settings.

## 5. Conclusions

The study’s results revealed that a wide range of barriers affected SVIs’ social participation. How the students experienced this phenomenon depended on various personal and environmental contextual factors. Personal factors, including poor self-image and self-belief, mistrust, a lack of independence, and poor communication and social skills, significantly impacted SVIs’ social participation experience. The interaction of personal factors and environmental challenges, such as attitudinal, sociocultural, and practical attributes, determined SVIs’ social participation. The results were discussed concerning the collectivist cultural context of the study setting and indicated the crucial role cultural orientation might play in influencing personal and environmental contextual factors and determining social participation. Interventions are needed to unblock wide-ranging barriers to social participation and meet positive health and wellbeing outcomes. More research is also needed to understand how social participation is crucial to giving life meaning and what it feels like to be socially ostracised in different cultural orientations beyond exploring barriers.

## 6. Limitations

We acknowledge the limitations of our study and note opportunities for future work. The findings of this qualitative inquiry from a small sample of participants raise questions about the findings’ generalisability. As such, future research should consider additional data from more SVI participants and stakeholders from school and community settings.

## Data Availability

Data are available on request due to privacy and ethical restrictions. The data presented in this study are available on request from the corresponding author. The data are not publicly available due to the privacy of the people who took part in this research.

## Appendix A

**Table A1:** Example Transcript: Semi-structured interview with SVIs–Student 1 (S1)–School A, Form 6, 2020.

Initial Codes from the Interview Data of SVIs	Categories	Emerged Themes & Subthemes
Interviewer (I): Hi (S1). How are you? Thank you for your willingness to participate in this study. We have time to talk?	Family income and education Environmental barrier (lack of information)	Family socio-economic status Inaccessibility of information
Participant student 1 (S1): I’m good, it’s okay. Yes, I have time, we can start.		
I: OK S1, can you tell me about the onset of your vision impairment?		
S1: It started when I was three.		
I: Do you have partial sight?		
S1: Yes, I’m partially sighted?		
I: Can you tell me about your family? (Where your family is, the size of the family, parental education, source of income …)		
S1: My family lives more than three hundred kilometers away from Addis Ababa. I don’t remember when my dad died. It was my mother’s responsibility to support the family. I’m the third child in my family. We are six altogether, four males and two females. My mother is not educated		
I: If you don’t live with your family, who provides you with support and how do you come here?	Environmental barrier (unstable resident area)	New social setting and lack of social resource
S1: My uncle brought me here when I was eight. With the help of him, I started to receive financial assistance from an organisation. I went to school too, and stayed with my uncle for five years. Due to financial problems, the organisation quit the support. I began to face a lot of challenges [sigh]. Anyway, I had to leave my uncle and family behind. After some troubles, I began to sell tickets to the national lottery agency and get some money. Sometimes some individuals who meet me doing this, encourage and support me in different ways. I have lived with three friends in a shabby house for three years now. We share our expenses; we keep our schooling. Now we have few friends in school	Environmental barrier (attitude)	Rejection
I: What kind of (extra-curricular) activities do you know that peers participate in School And the community? Do you have any experience of participating in these activities?	Environmental barrier (attitude) Environmental barrier (attitude)	Bullying Unequal treatment
S1: There’re a lot of clubs and activities in school that students voluntarily participate in. Additionally, in the community, there are also youth leagues, youth centers and the like that invite youth to participate. But, I don’t even know how to get involved in it		
I: What do you mean by ‘you don’t know how’? What’s the problem?	Environmental barrier (beliefs)	Social values as barrier
S1: I have never seen people invited to membership or participate. Maybe the invitation is written and posted on a noticeboard for those who can read.		
I: How is your social participation with your neighborhoods? Do you have friends and relationships with the community at various events?	Environmental barrier (estrangement)	Poor social capital Lack od confidence
S1: I have no social participation with my neighbors. I live in different new areas of residents in the city. I’m always an outsider to them. We don’t know each other. If there is some event in the family of our house renter and I’m invited, I would participate passively without interacting with any people there. In the neighborhood, I don’t have many friends. Most people will avoid you. Some young people make fun of you. It’s not easy to have many good friends. But fortunately also, I have a friend who encourages me to go with him and attend religious services. These kinds of people are medicine to this world. Their help is immense to make us feel good and participate in some areas of life.		
I: What does your participation look like in other areas of life in the community as leisure activities?	Personal barrier (lack of confidence) Personal barrier (being introvert) Environmental barrier (financial hardship)	Lack of communication skill Financial problem, practical limitation Sociocultural values as barrier
S1: I sell tickets to make my living and have little spare time. However, I enjoyed attending religious services. People have different approaches in the church, they have empathy. At home and in society, I’ve never been treated equally. My siblings and peers have greater value than me. There’s something wrong in this area. Look, most people do not question the way they think. They take for granted what they receive from society as true. Some educated people, including our teachers, are the same as the uneducated farmers in rural areas		
I: Do you mean you have poor social resources in all the areas you mentioned?	Environmental barrier (beliefs)	Transportation as practical barrier
S1: Yes, I have no such resources. I had already left my family for numerous social and economic reasons. I face similar experiences in the city too. My friends and I live on a social island.		
I: What do you mean ‘you live on an island within the city’?	Environmental barrier (mobility)	
S1: It’s obvious, the island is an isolated area from the larger landmass. I used this analogy to reveal that we also live in isolation with the community although we are not physically isolated.		
I: So how did you manage to get through this experience?	Environmental barrier (policy and institution)	Poor implementation of policy, practical challenge
S1: I trained myself to live alone. I see attaining an active social life is not easy for a person with vision impairment. So, I’m less interested in ensuring acceptance and participation.		
I: If you have such an unsatisfactory social participation experience, what’s the reason for this reality? How do you evaluate your share of the circumstances?		
S1: Let me begin with the second question. Frankly speaking, I’m shy and not sociable. I don’t know how to start communicating with people. Additionally, effective social participation requires the ability to freely move and identify places. These are all my problems. But, most of the challenges come from the outside. You know social relationships are bidirectional. It’s not only your interest, but also the acceptance of others that plays a major role. Sighted peers feel like we are a burden and try to avoid us as much as possible. I’m financially unstable and should refrain myself from such aspects. So, I don’t even have time to take part in the activities we mentioned earlier. However, even if we wish, you can’t force relationships. The traditional belief in the community is still abrasive. Moreover, you can’t overcome the transportation challenges in the city if you try to participate in some recreational activities, for instance. The government is not working as per policies and regulations. We always hear about these policies, but the realities are not changed.		

## Appendix B

**Table A2:** Participants’ responses and frequency of themes. SVIs interview responses: What barriers affect your social participation at home, in the community, and in school?

Themes	Student Response																	
	S1	S2	S3	S4	S5	S6	S7	S8	S9	S10	S11	S12	S13	S14	S15	S16	S17	
What barriers do affect their social participation?																		Total
Personal barriers																		
Poor self-image and personal belief	-	√	√	-	√	-	-	√	√	-	√	-	√	√	-	-	√	9
Endangered wellbeing	√	√	-	√	√	-	√	√	√	-	√	-	√	-	√	√	√	12
Mistrust	-	√	√	-	√	-	-	-	√	√	√	√	-	√	-	√	-	9
Deteriorated sense of independence	√	√	-	√	√	√	√	√	√	-	√	√	√	-	√	-	√	13
Lack of communication and social skills	√	√	√	√	-	√	√	√	√	√	-	√	√	-	-	√	√	13
Attitudinal																		
Unequal treatment	√	-	√	√	√	√	-	-	√	-	√	-	√	-	√	-	-	9
Overprotection	-	-	√	-	√	√	-	-	√	-	√	√	-	-	-	-	-	6
Peer rejection	√	-	√	-	-	√	√	-	-	-	-	√	-	√	√	√	-	8
Bullying & labelling	√	-	√	-	-	√	√	-	√	-	-	√	√	√	√	√	√	11
Low expectation and motivation	-	√	√	√	-	√	√	√	-	√	√	-	√	√	-	√	√	12
Sociocultural																		
Community beliefs and values	√	√	-	√	√	√	√	√	-	√	-	√	√	√	√	√	√	14
Lack of social support	-	√	√	√	-	-	√	√	√	-	√	√	-	√	√	√	√	11
New social settings and low social capital	√	-	√	√	-	-	-	√	√	-	-	√	-	√	√	-	-	8
Practical																		
Family status	√	√	-	-	-	-	√	√	√	√	-	-	√	√	-	-	√	9
**Lack of access to adaptive equipment**	-	√	-	-	-	√	√	-	√	-	-	√	-	-	-	√	√	7
Weak teamwork & collaborative practices	-	-	-	√	√	-	√	-	-	-	√	-	-	√	√	-	-	6
Physical and informational accessibility issues	√	√	√	√	√	-	√	√	√	-	√	√	-	√	√	√	√	14
Transportation	√	√	-	-	-	√	-	-	-	√	√	-	-	-	-	√	√	7
Financial scarcity	√	√	√	√	-	√	-	-	√	√	√	-	√	√	√	√	√	13
Policy and practice	√	√	-	-	√	√	-	-	-	-	√	√	-	√	-	√	√	9
**Individual sharing, out of the 20 themes**	13	14	12	12	11	12	12	10	14	7	13	12	10	13	11	13	14	
